# Digital competence using the example of executives in residential care facilities in Germany—a comparison

**DOI:** 10.3389/frhs.2024.1372335

**Published:** 2024-05-21

**Authors:** Stefanie Liebl, Tim Tischendorf, Michel Hummel, Lydia Günther, Tom Schaal

**Affiliations:** Faculty of Health and Healthcare Sciences, University of Applied Sciences Zwickau, Zwickau, Saxony, Germany

**Keywords:** digital competence, inpatient care facilities, DigComp, online survey, cross-sectional survey

## Abstract

**Background:**

Change and progress through digitalisation is also becoming increasingly important in the field of professional care and the associated increasing demands on the skills of nursing staff. The European Union considers digital skills to be one of the eight key competences for lifelong learning. At present, few reliable statements can be made about the status of digital skills in professional nursing care in Germany. The aim of this study was to map the current status of digital competences of executives in full inpatient care facilities in Germany and to identify possible differences to reference values of academics.

**Methodology:**

This survey is based on a Germany-wide cross-sectional survey in full inpatient care facilities (*N* = 8,727). The survey instrument Digital Competences Framework (DigComp 2.2) according to the European Union's reference framework was used as the basis for recording the digital competence characteristics. The statistical analysis was descriptive and inferential (*t*-test, two-sided, *p* < 0.05).

**Results:**

Out of 15 items across five dimensions, significant differences for nine items can be determined. The competence levels of the participating managers from the full inpatient care facilities were lower compared to the reference sample.

**Discussion:**

In order to be able to counter the skills discrepancy shown by the study in the future, it is of central importance to deepen knowledge and skills in the area of digitalisation in the care context.

## Introduction

Technology and technologisation are reflected in almost every area of everyday life. The number of Internet users worldwide is rising continuously. In 2022, the number of users, also known as “onliners”, was around 5.3 billion, an increase of 2.9 billion in the last ten years (1). In this context, the question arises as to how digitalisation is defined. Generally speaking, it is the change in the electronic storage, networking and processing of information and, in particular, the integration of technologies into work processes (2). Digital skills are one of the eight key competences of lifelong learning defined by the European Union (EU) (3). Digital literacy encompasses the safe and critical use of digital technologies, which are used for information, communication and problem-solving strategies in all areas of life (4). In this context, however, a study by the EDCL Foundation (2014) draws attention to a fallacy regarding the so-called “digital natives”. Simply growing up with the technological progress of computers, mobile phones and other technologies is no indication of a person's digital competence. There is the possibility of the incompletely acquired ability to use these technologies safely and efficiently (5). Contrary to the assumption that young people are automatically exempt from acquiring digital skills, there is a new digital divergence between the actual skills of the digital lifestyle and the skills for a digital workplace, which should not be disregarded.

The constant change and progress of digitalisation, particularly in the nursing context and the associated growing demands on the skills of nursing staff, are becoming increasingly important (6). In recent decades, a dynamic process of lifelong learning has emerged, which is reflected in the general lengthening of educational pathways and the often changing occupational fields within a profession (7). In addition to the numerous opportunities for society and, in particular, the professional care sector, the use of technology also contains risks. Digitalisation still raises many unanswered questions in this field of operation. When it comes to digitalisation in the healthcare sector, there are various associations that are linked to different elements of modern healthcare. These include algorithms, the processing of large amounts of data for complex medical conditions, state-of-the-art medical devices and surgical robots. These associations are undoubtedly correct, as these aspects play an increasingly important role in modern healthcare, as does their differentiation (8). Digital transformation refers to the conversion of data, documents and processes from analogue to digital. Digitalisation is the transformation of business processes (9).

The aim of this study was to map the current status of digital competences of executives in full inpatient care facilities in Germany and to identify possible differences to reference values of academics. At present, few reliable statements can be made about the level of digital skills in professional care. This is primarily due to a lack of meaningful data. The studies available indicate that the sector is lagging behind others in this area (6). Compared to the leading Scandinavian countries Finland and Denmark in the European ranking of the digital economy and society in 2022, Germany is in 13th place (10). Only a few assistance technologies are currently being used in German hospitals and care facilities (11). The efficient processing of health data and the establishment of comprehensive data-based medicine are essential elements of a modern healthcare system. However, Germany is still a developing country in this context. This is confirmed by a recent study by the Organisation for Economic cooperation and Development (OECD). In an international comparison, Germany only ranks third to last in terms of accessibility and linking of health data. The Scandinavian countries in particular are leading the way (12). On December 2023, the German Bundestag passed the Digitalisation Act (DigiG) and the Health Data Usage Act (GDNG) to drive forward digitalisation in the healthcare sector. The latter aims to improve the use of healthcare data.

McKinsey & Company tracked the progress of the healthcare industry using around 30 indicators, including the degree of digitalisation of doctors’ practices and hospitals as well as the acceptance of e-health tools for patients. The expansion of the telematics infrastructure (TI), to which almost 99 per cent of all pharmacies and doctors’ surgeries are now connected, is also having a positive impact. Nevertheless, two thirds of connected practices cite technical problems that occur weekly or even daily in everyday practice as the reason for the digitalisation of the healthcare system. The activation of electronic patient records is also progressing slowly. Only one per cent of those with statutory health insurance currently have an activated electronic patient file (13). The reason for this is the seemingly less lucrative cost-benefit ratio (11). In the coming years, however, an upswing in the field of information and communication systems, as well as robotics and technical assistance systems is predicted due to an improvement in the cost-benefit ratio and an increase in suitability for everyday use (11). It can be assumed that continuing technological progress and the increased use of assistive technologies will have an impact on the everyday work of caregivers, which requires the training of digital skills (11).

In a survey conducted by the ifo Institute (Leibniz Institute for Economic Research) in May and June 2021, 56% of respondents across Germany stated that digital and media skills are very important for the future of society. A further 36% rated them as somewhat important. The global megatrends of globalisation, artificial intelligence, health and digitalisation should not be ignored to illustrate the ever-increasing relevance of digital skills for Germany's participation in international competition (14). In view of the ever-increasing demands placed on nursing staff in the areas of digital documentation, data management and the use of diagnostic and decision-making tools, further research in this area is urgently required (15).

The present study focuses on the digital competence of executives in residential care facilities in Germany with regard to data processing and evaluation, communication and cooperation, content creation, security and problem solving. A manager in a care facility is understood as a person who holds a managerial position within the organisation. As a minimum, they are entrusted with personnel management in a defined area of this organisation and usually also have budget and material responsibility. As executives in care facilities also have access to personal data and have to deal with complex management tasks, it can be assumed that they must also have digital competences in accordance with the EU Framework.The DigComp 2.2 questionnaire forms the basis for this survey regarding digital competence.

## Methodology

The nationwide cross-sectional survey in full inpatient care facilities was realised with the help of the online platform SoSci-Survey (16). Email addresses from 8,727 inpatient care facilities were included, which were provided as a data set by the AOK-Bundesverband for scientific purposes (17). No phone calls were Made, only a reminder about the survey was sent. The management of the care facility was able to distribute the survey to the relevant employees. The AOK Care Navigator contained gaps, meaning that 8,727 of the 11,358 fully inpatient care facilities in Germany in 2021 could be taken into account. Nationwide, North Rhine-Westphalia has the most fully inpatient care facilities in Germany with 2,244, followed by Baden-Württemberg with 1,536 and Bavaria with 1,504. Bremen has the lowest number of facilities nationwide with 97 fully inpatient care homes (18).

Invitations to participate in the online survey were sent via the mail merge function of SoSci-Survey. On the landing page of the online survey, consent to participate had to be actively given by clicking on it before accessing the actual questionnaire. If this was not given, participants were redirected to the end of the questionnaire. The survey was conducted in compliance with the applicable data protection regulations, with a comprehensive data protection concept available on the landing page. The survey period was from March 23rd to April 30th 2023 and a reminder email was sent on April 18th. Descriptive and inferential statistical analyses were carried out using IBM SPSS Statistics 29 (*t*-test, *p* < 0.05, two-sided).

The DigComp 2.2 questionnaire according to the EU reference framework was used as the basis for recording the digital competence characteristics. It is constructed according to Krempkow (2022) is a theoretically and empirically based survey instrument for recording digital competences in accordance with the DigComp 2.1 reference framework of the EU (19), which was used in the KaWuM-Survey 2 trough out Germany in 2022 (20). As part of this survey, reference data was collected from over 1,200 science managers and around 7,000 students from several large universities in Germany across all subject groups. Science managers organise, control and design the relevant processes at universities. These include, for example, research funding, personnel development, organisational development and controlling.

The DigComp 2.2 was also used in the survey of the digital competences of executives from inpatient care facilities in order to ensure appropriate comparability of the data on the basis of a standardised measurement instrument. A comparison of the executives of the inpatient care facilities with the sample of the KaWuM survey is appropriate, as it can be assumed that the executives of the inpatient care facilities have a comparable level of academic competence and are qualified in terms of leadership in a similar way to the scientific managers.

With 15 items, the DigComp 2.2 questionnaire construct covers all five dimensions (subscales) of digital competences according to DigComp 2.1. The subscales include (1) data processing/evaluation, (2) communication/cooperation, (3) content creation, (4) security and (5) problem solving. They are answered on a five-point response scale (1 = to a very great extent to 5 = not at all) (10). In addition, an open knowledge test question was asked in order to determine the extent of some suspected tendencies to overestimate oneself in relation to the self-assessment of search strategies within the subscale of data processing/analysis. A possible overestimation with regard to reliable research strategies on the internet does not exist if the research results are systematically analysed from various sources in order to ensure a broad perspective and a comprehensive assessment. This includes the consideration of specialised literature, research studies and expert opinions in order to arrive at a well-founded conclusion that adequately reflects the complexity of the topic. A suitable approach would be, for example, the creation and application of search strings using Boolean operators, truncation, synonyms and the definition of inclusion and exclusion criteria (19).

The 15 items surveyed on digital skills were not normally distributed (Shapiro-Wilk test). Only mean values were available for the reference values of the KaWuM-Survey 2, which is why the *t*-test based on medium-values was used in this study. Simulation studies have shown that the *t*-test is largely robust to violations of the normal distribution assumption and has proven to be suitable for the following analysis (21).

## Results

Of 8,727 emails, 830 could not be delivered shown by an error message. The link to the survey was clicked 830 times (click rate of 9.5%). 290 people took part in the survey, 184 of whom completed the questionnaire. The net response rate was 2.1%.

One hundred seventy-five out of 290 participants provided information on their function in the care facility. Most of the responses in terms of age were in the 40–49 age range and 50–59 age range (Table 1).

**Table 1:** Current role in the organisation by age group.

		Function					Total
		Management	Facility management	Nursing service management	Quality management	Other	** **
Age	18–29 years	0	2	1	1	7	11
	30–39 years	2	8	14	1	12	37
	40–49 years	1	17	19	3	16	56
	50–59 years	5	17	9	4	15	50
	60–69 years	2	10	5	0	4	21
Total		10	54	48	9	54	**175**

**Figure 1:**
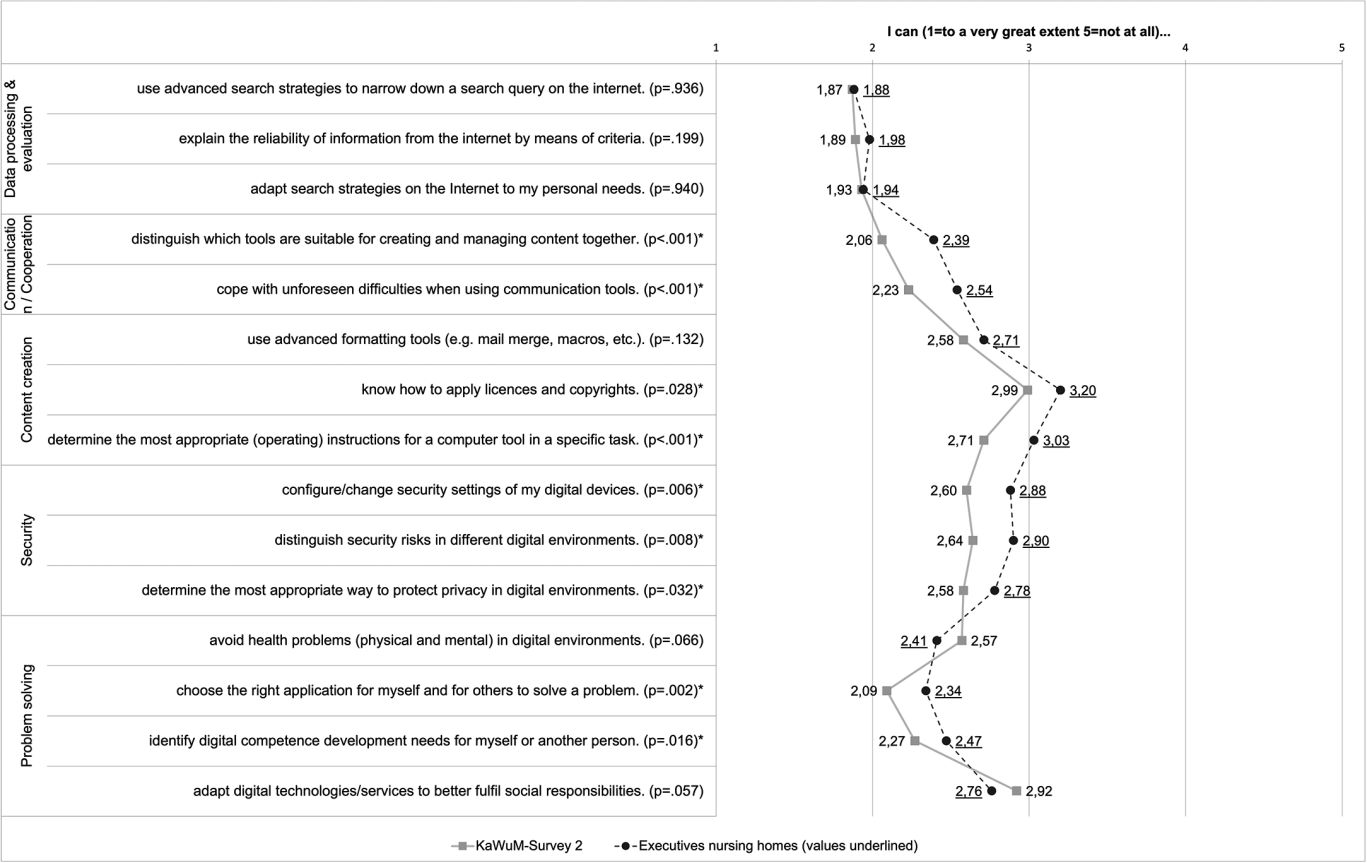
Mean values of the digital competences recorded (*difference statistically significant).

The management is responsible for the strategic direction, financial planning and legal affairs of the facility. It bears overall responsibility for the economic success and long-term development of the care home. The care home management is responsible for the operational management of the care home. They coordinate day-to-day operations, monitor the quality of care and support, are the point of contact for residents and relatives and take care of organisational matters such as personnel management and compliance with standards. The nursing service manager is responsible for the nursing area within the care home. They are responsible for the professional management of the nursing staff, the planning and implementation of care measures, ensuring the quality of care and compliance with legal requirements and guidelines.The “other” category included, for example, social services or occupational therapy. The average work experience (*n* = 184) was 11.4 years (SD ± 9.7). 84.2% (*n* = 154) of the participants who answered the question on gender (*n* = 183) were female, 15.8% (*n* = 29) were male. The facilities in which the participants worked (*n* = 163) mostly had 50–99 (38%; *n* = 62) or 1–49 (27.6%; *n* = 45) care places. 61.7% (*n* = 108) of the participants classified their facility as non-profit, 29.7% (*n* = 52) as private and 8.6% (*n* = 15) as public (*n* = 175). The federal states with the highest response rate (*n* = 182) were Bavaria (*n* = 40), Saxony (*n* = 40) and North Rhine-Westphalia (*n* = 39). Of the total of 15 items of the five subscales of the DigComp 2.2 construct, significant differences were found in nine items (Figure 1).

No significant differences were found with regard to the first dimension of data processing/evaluation. In addition, an open knowledge question was asked as free text in connection with the first dimension of data processing/evaluation. This included the question of which search strategies the respondents use to search for reliable information on the Internet. Of the 234 respondents, a total of 73 answered this question. The most frequently given answer was Google (*n* = 29). In addition, keyword searches (*n* = 19), strategies customised to the question or problem (*n* = 5) and Boolean operators (*n* = 3) were cited as search strategies. A total of 17 answers could not be categorised. These include “no answer” (*n* = 2) and “secure websites” (*n* = 1) or “no internet on site” (*n* = 1).

In the communication/cooperation and security subscales, on the other hand, there were significant deviations from the DigComp 2.2 reference values in all items. With regard to the content creation subscale, significant differences were found in the statements on knowing how to apply licenses, copyrights and determining appropriate (operating) instructions for a specific task. In the fifth dimension, problem solving, significant differences became apparent in the ability to select the right application for themselves and others to solve a problem and to determine the need for further development of digital skills for themselves and others.

The mean deviations were above the reference values in 13 of the total of 15 items among the respondents from the care facility managers, which indicates lower digital skills among the participants compared to the KaWuM survey sample. Due to the non-parametric properties of the variables, the Kruskal-Wallis test was statistically calculated for differences between the items of DigKomp 2.2 according to Krempkow in relation to the stated activity of the executives of inpatient care facilities participating in our survey. No significant differences were found between the individual activity statements in relation to the DigKomp 2.2 items.

## Discussion

In relation to the five dimensions of DigComp 2.2, the communication/cooperation and safety subscales show the greatest differences to the reference values of this study. In both dimensions, the reference values for digital competence in the reference sample of the KaWuM survey were significantly better than for participants in care facilities in this survey. With regard to the communication/cooperation dimension, early involvement and the ability to engage with digital team tools during (academic) training can make a decisive contribution. Related to this, the study by the EDCL Foundation (2014), among others, makes it clear that a differentiated distinction should be made between “digital lifestyle” and “digital workplace skills” (5). These “digital lifestyle skills” are not the skills required, for example, to get a job, negotiate with authorities or manage healthcare. The latter skills require formal and structured training (5).

Safe, critical and responsible use of and engagement with digital technologies requires a profound level of security competence within digital skills (3). Particular attention should therefore be paid to the significantly lower competence estimates of respondents from nursing care facilities in the security dimension. Medical data, for example, is particularly worthy of protection in the healthcare sector. The competence area of security can be divided into four individual competences, which should be considered in curricular developments of training and further education programs. These include the protection of devices, personal data and privacy, the environment as well as health and wellbeing (3).

Based on the open knowledge question in the data processing/evaluation dimension on search strategies and the free text answers provided, there is a potential overestimation of the digital skills of care staff, which is confirmed by the most frequently specified response to the use of the search engine “Google”. In this context, the work of Krempkow et al. 2022 can be used to categorize the results of the knowledge question. In his study, the students surveyed were found to overestimate the search strategies they use on the Internet to customise their personal searches (22). These outcomes emphasise the importance of targeted promotion of digital competencies in the healthcare sector to ensure that nursing staff can access digital resources effectively and efficiently. The promotion should therefore not be limited to basic, advanced and further training, but should already be an integral part of nursing training.

Measured by the mean values, the reference values for the two items “I know how to apply licenses and copyrights” and “I determine the most appropriate (operating) instructions for a computer tool in a specific task” in the content creation dimension were statistically significantly better than for the participants in the care facilities. The individual skills in the content creation dimension include developing digital content, using and editing third-party digital content, knowledge of copyright as well as free licenses and programming. This suggests that those outside the care sector may have more advanced digital competences in terms of content creation, which has important implications for digital training and support for nurses. There may be a need to provide targeted training programmes or resources to ensure that nurses have the necessary skills to create, use and manage digital content effectively, which could ultimately improve the quality of nursing care and meet the demands of an increasingly digitalised healthcare landscape (14).

In the fifth and last dimension of the digital competencies survey, statistically significant differences were found, particularly for the items “I can choose the right application for myself and for others to solve a problem” and “I can identify digital competence development needs for myself or another person”. Digital competences for problem solving represent an end point of digital competences that builds on previous basic competences (3). This is another crucial aspect that should be taken into account in the systematic development of digital skills in the healthcare sector. If basic skills in dealing with digital technologies are insufficiently developed, this leads to further competence deficits in the search for solutions to problems. Care service managers should receive targeted training on individual skills with regard to the careful use of time resources. Specific skills, such as data management, should be passed on to internal experts such as data protection officers or IT officers. They can also act as multipliers within the institution to ensure knowledge management within the institution. It might also be possible to offer individual courses or workshops for nursing staff who have already been working in nursing for several years, which could be offered in addition to the basics of nursing training. The introduction of digital innovations in care facilities can be analysed through the NASSS framework, taking into account factors such as the acceptance of the technology by care staff, the availability of resources for training and support, and the integration of digital systems into existing workflows. The initiation of digital competences among care professionals therefore requires targeted training and support to ensure the successful use and integration of digital tools into care practice and thus promote sustainable implementation.

Becka et al. make it clear that with the increasing importance of digital technology in the nursing context, the work processes specific to the profession will change fundamentally. As a result, more training should be provided on the competences relevant to the respective changing work processes. For example, digital skills are becoming increasingly important for more highly qualified and managerial nursing staff, whereas communication and cooperation should be prioritised for nursing staff in non-managerial positions (15).

In the context of Becka et al., it can be summarised that the digital competence of full inpatient care facilities in Germany is an important factor in improving the quality of care and meeting the increasing demands of the ongoing structural change in Germany (15). The results of the study indicate that the respondents of the facilities in question have lower digital skills than the reference values from the KaWuM survey. This emphasises the importance of promoting digital competencies both during the further training of care professionals as well as during the education of these professionals. It is crucial that all stakeholders in the healthcare sector work together to drive forward digitalisation and use their respective potential to actively shape progress together. The education sector in particular should focus on promoting digital skills development in the training of specialists at vocational medical schools. In addition to the education sector, politicians also have a duty to revise the Nursing Professions Act regarding the lack of digital skills. This relevant problem is already becoming the subject of various projects by the Federal Institute for Vocational Education and Training (BIBB) in an attempt to develop the skills of teachers and future nurses. The Digital Competences through Participation (DigiK-Part) project has the goal of establishing the digital competences of teachers and future teachers at nursing schools and universities. In addition to deepening knowledge in the field of digitalisation in the nursing context and the latest technologies, special attention should be paid to the consequences of digital technologies in the continuing education program, focusing not only on nursing processes, but also on the consequences for the work of nurses (14). The topics should focus in particular on acceptance, satisfaction and stress on both a psychological and physical level (14). In an internet and literature search by Nüßlein and Schmidt (3), it was found that, compared to its European neighbors, there are few to no offers in the sense of a 3-stage learning model in the area of DigComp 2.1 in Germany. There is an urgent need to expand the programs and increase their presence (3). A suitable political framework and the anchoring of digital skills in the EU key competences, which support companies in refinancing measures in the course of digitalisation, should be further promoted in order to make it more attractive for employers to make use of them.

## Limitation

The online survey was a cross-sectional survey, which is why it is not possible to map developments over time. The sample of inpatient care facilities in Germany was based on data from the AOK Care Navigator (16). Depending on the federal state, there were gaps in the contact data records. On the one hand, e-mail addresses of the facilities were missing. On the other hand, contact details were not up to date. As a result, only 8,727 of the existing 11,400 facilities could be contacted which leads to an incomplete data collection.

Personalised links were not used, as these would have lost their validity if forwarded internally within the facilities. This meant that multiple participation could not be excluded technically. As the functional email addresses from the AOK Care Navigator were used for recruitment, it can be assumed that the executives accepted the email and that multi-level forwarding to nursing staff was not favoured. As a result, however, the Other notification as an activity showed that third parties also had access to the questionnaire. However, a further review showed that this had no influence on the mean values or the significance.

The questionnaire category of digital competences was based on the theoretically and empirically based survey instrument DigComp 2.2 construct according to Krempkow (2022). It should be noted here that the competence assessments may be subject to limitations from the outset due to self-assessment bias (20). The uncertainties identified in this context in the area of digital skills can be used as thematic focal points in the further work and should be investigated more in-depth. With regard to the dropout rate of the questionnaire, social desirability and survey fatigue can be assumed as possible factors.

Taking into account the response rate of 2.1% the high drop-out rate can be attributed to the complexity of the question or a lack of interest. Social norms and survey fatigue were also discussed as possible factors that contributed to the cancellation of participation. For a better understanding, an overview of the drop-out rate of the study has been included in the appendix.

## Data Availability

The raw data supporting the conclusions of this article will be made available by the authors, without undue reservation.
