# Supplementary material for: Digital competence using the example of executives in residential care facilities in Germany—a comparison
Source: Front Health Serv. 2024 May 21;4:1372335. doi: 10.3389/frhs.2024.1372335 (PMC11148298; doi:10.3389/frhs.2024.1372335)
Supplement: Supplementary file 1 [file Datasheet1.pdf]

The following is an assessment of your skills in dealing with digital media and technologies in accordance with the EU's Digital Competence Framework (DigComp2.1).

---

1. To what extent do you currently have the following skills?

Data processing and evaluation

**I can ...**	even not				to a very high degree
	0	1	2	3	4
use advanced search strategies to narrow down a search query on the Internet.					
explain the reliability of information from the Internet by means of criteria.					
adapt search strategies on the Internet to my personal needs.					

What search strategies do you use for this?

please enter

Communication and cooperation

**I can ...**					
distinguish which tools are suitable for creating and managing content together.					
cope with unforeseen difficulties when using communication tools.					

Content creation

I can ...

use advanced formatting tools (e.g. mail merge, macros, etc.).

know how to apply licences and copyrights.

determine the most appropriate (operating) instructions for a computer tool in a specific task.

Security

I can ...

configure/change the security settings of my digital devices.

distinguish security risks in different digital environments.

determine the most appropriate way to protect privacy in digital environments.

Problem solving

I can ...

avoid health problems (physical and mental) in digital environments.

choose the right application for me and for others to solve a problem.

identify digital competence development needs for myself or another person.

adapt digital technologies/services to better fulfil social responsibilities.

2. **What is your current role in your organisation?**

Management Centre

management Nursing

service management

Quality management

Other (please enter):

Not specified

3. **How many years of professional experience do you have in the above-mentioned position?**

years

4. **How old are you?**

18-29 years

30-39 years

40-49 years

50-59 years

60-69 years

70-79 years

80 years and older

Not specified

5. **Which gender do you categorise yourself as?**

male female

diverse

Not specified

6. **How many fully inpatient care places does your facility have?**

0-49

50-99

100-149

150-199

200 or more

Not specified

7. **Which organisation runs your facility?**

non-profit public

private

Not specified

8. **In which federal state is your organisation located?**

Schleswig-Holstein
Hamburg
Lower Saxony (Niedersachsen)
Bremen
North Rhine-Westphalia (Nordrhein-Westfalen)
Hesse (Hessen)
Rhineland-Palatinate (Rheinland-Pfalz)
Baden-Württemberg
Bavaria (Bayern)
Saarland
Berlin
Brandenburg
Mecklenburg-Vorpommern
Saxony (Sachsen)
Saxony-Anhalt (Sachsen-Anhalt)
Thuringia (Thüringen)
