# Supplementary figures and images for: Digital competence using the example of executives in residential care facilities in Germany—a comparison
Source: Front Health Serv. 2024 May 21;4:1372335. doi: 10.3389/frhs.2024.1372335 (PMC11148298; doi:10.3389/frhs.2024.1372335)

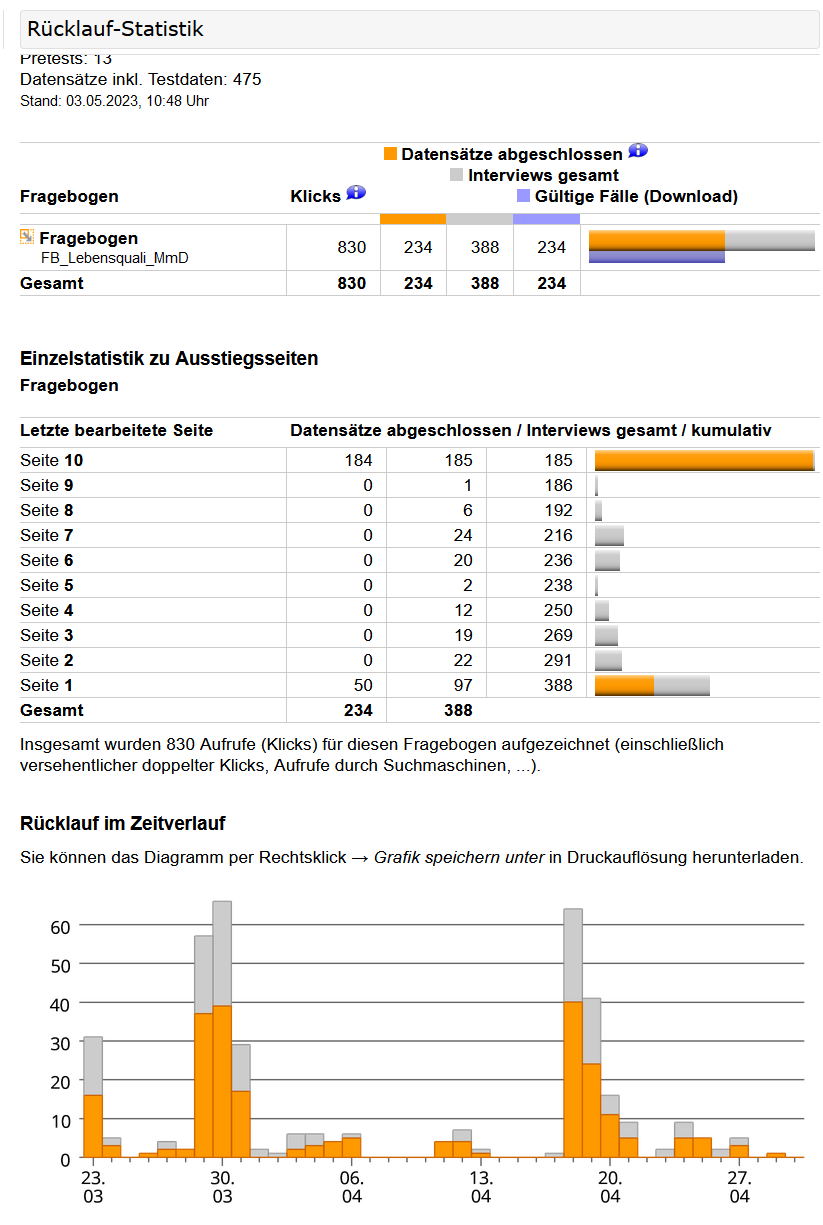

Supplement: Supplementary file 2 [file Image1.png]
